# The parasitic worm product ES-62 normalises the gut microbiota bone marrow axis in inflammatory arthritis

**DOI:** 10.1038/s41467-019-09361-0

**Published:** 2019-04-05

**Authors:** James Doonan, Anuradha Tarafdar, Miguel A. Pineda, Felicity E. Lumb, Jenny Crowe, Aneesah M. Khan, Paul A. Hoskisson, Margaret M. Harnett, William Harnett

**Affiliations:** 1 Strathclyde Institute of Pharmacy and Biomedical Sciences, University of Strathclyde, Glasgow, G4 0RE UK; 2 Institute of Infection, Immunity and Inflammation, University of Glasgow, Glasgow, G12 8TA UK

## Abstract

The human immune system has evolved in the context of our colonisation by bacteria, viruses, fungi and parasitic helminths. Reflecting this, the rapid eradication of pathogens appears to have resulted in reduced microbiome diversity and generation of chronically activated immune systems, presaging the recent rise of allergic, autoimmune and metabolic disorders. Certainly, gastrointestinal helminths can protect against gut and lung mucosa inflammatory conditions by modulating the microbiome and suppressing the chronic inflammation associated with dysbiosis. Here, we employ ES-62, an immunomodulator secreted by tissue-dwelling *Acanthocheilonema viteae* to show that helminth-modulation of the gut microbiome does not require live infection with gastrointestinal-based worms nor is protection restricted to mucosal diseases. Specifically, subcutaneous administration of this defined immunomodulator affords protection against joint disease in collagen-induced arthritis, a mouse model of rheumatoid arthritis, which is associated with normalisation of gut microbiota and prevention of loss of intestinal barrier integrity.

## Abstract (web summary)

Gastrointestinal infection with parasitic helminths can protect against mucosal diseases via impacting on the microbiome. Here the authors show that ES-62, a product secreted by a tissue-resident helminth modulates the host gut microbiome to protect against inflammatory arthritis in a mouse model.

## Introduction

Parasitic helminths (worms) have evolved to modulate host immune and tissue repair responses to promote their survival by limiting inflammation that would otherwise drive their expulsion and cause pathology^1^. The recent eradication of helminths (and other pathogens) appears to have resulted in over-activated immune systems and this provides a rationale for the increasing prevalence of allergic and autoimmune inflammatory disorders, as well as contributing to the rise in obesity and associated comorbidities^2–4^ in developing and urbanised countries. Although genetic studies have identified gene variants associated with various inflammatory diseases, these alone do not appear to be strong risk factors, integration with environmental factors being required to trigger disease. Recognition of this has focused interest on the role of the microbiota^3,5^ and hence, on how helminths may regulate this in health and disease^6,7^. Indeed, commensal bacteria and gastrointestinal (GI) helminths appear to have evolved to reciprocally regulate the gut microbiome^8^ to homeostatically maintain immune system function^2^. Thus, GI helminths can induce regulatory responses to limit inflammation and promote intestinal barrier integrity, while intestinal bacteria play an essential role in training the immune system by impacting on stem and progenitor cells^3,5^. Certainly, there is increasing evidence from animal models that protection afforded by GI helminth infection against mucosal inflammatory disorders like asthma, inflammatory bowel disease and coeliac disease, involves modulation of the gut microbiota^2,6,7^.

Nevertheless, gut, lung or oral dysbiosis has also been implicated in the aetiology of a wide range of autoimmune diseases, including musculoskeletal pathologies like rheumatoid arthritis (RA) and systemic lupus erythematosus (SLE)^9,10^. Whether the protection afforded by GI helminths against these disorders similarly involves interaction with the microbiome is not clear but infection with *Heligmosomoides polygyrus* and *Trichuris muris* can result in increases in *Lactobacillaceae* and decreases in *Prevotella* species^2,11,12^, commensals reported to be dysregulated in RA patients^9,10^. In any case, helminth-mediated protection against autoimmune disease is not limited to GI-tract parasites, with particularly striking examples of this involving filarial nematodes preventing development of RA^13^ and SLE^14^. However, it is unclear whether tissue-resident or blood-borne parasitic worms can mediate these effects via modulation of the host microbiome and if so, which mechanisms they utilise.

That helminths can ameliorate chronic inflammatory disorders has often been attributed to their capacity to excrete or secrete molecules (ES) that exert immunoregulation^2^. Amongst the best characterised ES products is ES-62, a phosphorylcholine (PC)-containing glycoprotein secreted by the filarial nematode *Acanthocheilonema viteae* that we have shown to prevent initiation and progression of pathology in mouse models of certain allergic (asthma, contact dermatitis) and autoimmune (RA, SLE) inflammatory diseases^1,2,15–20^. Collectively, our studies have identified a unifying mechanism of action that allows effective protection irrespective of the inflammatory phenotype: thus, by subverting TLR4 signalling to downregulate aberrant MyD88-responses, ES-62 homeostatically resets the regulatory:effector immune cell balance, primarily to restore levels of IL-10^+^ regulatory B cells and suppress pathological IL-17-driven inflammation^1,2,15–21^. In both experimental models of RA and human disease, perturbation of the microbiota has been shown to disrupt the balance of pathogenic Th17 cells and the counter-regulatory Bregs and Tregs that act to homeostatically resolve inflammation^9,10,22^. Thus, our aim here was to investigate whether the anti-inflammatory actions of ES-62 reflected an ability to impact on the microbiome. We now show that whilst joint disease in the collagen-induced arthritis (CIA) mouse model of RA is preceded by disturbance of the gut microbiome with accompanying intestinal inflammation and loss of barrier integrity, ES-62 acts to normalise the microbiome and maintain gut health. Furthermore, we report that prophylactic depletion of the gut microbiota with broad-spectrum antibiotics (ABX) reduces the consequent severity of arthritis in mice undergoing CIA and in addition, reduces the level of protection afforded by ES-62. These data therefore indicate that a normalised microbiome is required for the full induction of the immunoregulatory actions of ES-62.

## Results

### ES-62 normalises the gut microbiome in protecting against CIA

ES-62 ameliorates CIA in terms of articular score and frailty, maintaining grip strength at a similar level to that of healthy, Naive (not subjected to CIA) DBA/1 mice (Supplementary Figure 1). Commensal bacteria have increasingly been proposed to contribute to RA pathogenesis^9,10^ and decline in grip strength during ageing has been associated with changes in the gut microbiome^23^. In addition to being an indicator of frailty, grip strength is a predictor of a wide range of adverse health outcomes^24^, e.g., cardiovascular disease, which RA patients are at increased risk of developing^25^ and that are impacted by the microbiome^9,26^. Thus, to address whether ES-62-mediated protection reflects modulation of the gut microbiota, a shotgun metagenomic approach was used to profile bacteria populations present in the intestines of CIA mice. Initiation of RA (and CIA) pathogenesis is associated with disruption of the balance of effector:regulatory immune cells and so we characterised the bacterial changes pertaining during established arthritis in the ileum and colon; intestinal sites where the microbiome and the metabolic microenvironment play key roles in shaping Th17 and regulatory immune responses^22,27,28^. As there is well-documented variation in the microbiome amongst individuals due to a range of environmental factors, we adopted the strategy of pooling samples from mice with representative disease scores to minimise variation due to a factor we could control for, namely disease severity: specifically, we focused on those samples associated with a well-established severe level of disease in CIA mice (articular score: 7.00 ± 0.91) and clear protection against arthritis in ES-62-treated CIA mice (articular score: 0.50 ± 0.33) over three independent experiments.

**Fig. 1:**
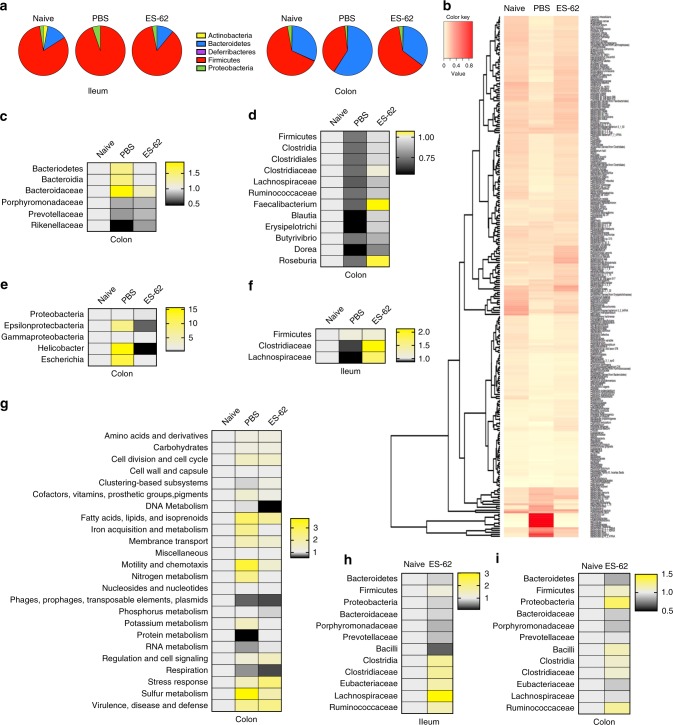
ES-62 normalises the microbiome during CIA towards a Naive phenotype. The composition of bacterial phyla present in the ileum and colon of Naive, PBS and ES-62-treated CIA animals, presenting proportion values as pie charts (**a**) from a single representative experiment using pooled samples from three mice in each condition. **b** Heatmap analysis of all bacteria present in the colon of Naive, PBS and ES-62-treated CIA animals from this representative model (articular scores; naive, 0; PBS-CIA, 6.3 ± 2.6; ES-62-CIA, 0; *p < 0.05 for PBS-CIA versus Naive and ES-62-CIA mice) is shown. The profile of bacterial abundance, is provided in Supplementary Table 1 for clarity. **c**–**g** Metagenonic analysis was performed on three independent models: in each model, samples from three representative mice/group were pooled in each individual experiment and the mice selected exhibited the following articular scores: Naive, 0; PBS-CIA, 7.00 ± 0.91; ES-62-CIA, 0.50 ± 0.33, where ***p < 0.001 for the PBS-CIA versus Naive and ES-62-CIA cohorts and *n* = 9 mice for all groups over the three independent models. Statistically significant changes (two-way ANOVA with LSD Fishers multiple comparisons) observed over the three independent experiments (mean ± SEM, *n* = 3) between the PBS-CIA and ES-62-CIA groups in Bacteroidetes (**c**; Bacteroidaceae; PBS versus Naive or ES-62, p < 0.05), Firmicutes (**d;** Clostridiales; PBS versus Naive, *p* < 0.01 and PBS versus ES-62, *p* < 0.05) and Proteobacteria (**e**; Epsilonproteobacteria; PBS versus Naive or ES-62, *p* < 0.05) in the colon and Firmicutes in the ileum (**f;** Clostridiaceae; PBS versus ES-62, *p* < 0.05) as well as functional metagenomics of the colon (**g;** Phages; Naive versus PBS, *p* < 0.01, Protein metabolism; PBS versus Naive or ES-62, *p* < 0.01) are presented as heatmaps with changes in bacterial populations in PBS- and ES-62-treated CIA animals normalised to Naive controls. Ileum (**h**) and colon (**i**) faecal matter from Naive or ES-62-treated Naive animals was analysed for changes in bacterial populations in a single experiment using samples pooled from three animals per group and displayed as heatmaps, where the ES-62-treated samples are normalised to the Naive controls

An overview of the microbiota at the phylum level shows substantial changes between healthy Naive mice and those with arthritis (PBS; Fig. 1a). Firmicutes and Bacteroidetes are the predominant phyla in all groups, but CIA mice in particular, exhibit outgrowths of Firmicutes and Proteobacteria in the ileum, whereas they demonstrate decreased levels of Firmicutes with a compensatory outgrowth of Bacteroidetes, in the colon (Fig. 1a). ES-62 essentially helps maintain the healthy microbiome diversity of Naive mice, which was reduced in CIA mice (Fig. 1a). Deeper analysis illustrates the differential diversity signatures of healthy and arthritic mice, as well as the impact of ES-62 on a global scale (Fig. 1b; Supplementary Table 1). As a consequence of our pooling strategy, we have refrained from discussion of species-level changes as large-scale population studies would be required to address this with complete confidence. Nevertheless, drilling down on the modulation of the Gram-negative Bacteroidetes phylum reveals differential signatures throughout each of the predominant *Bacteroides*, *Porphyromonas* and *Prevotella* genera and the *Rikenellaceae* family between the colon contents of Naive and CIA mice, identifying those normalised by exposure to ES-62 (Fig. 1c). Similarly, in spite of the fact that differential profiles amongst the groups were observed throughout major genera (e.g., *Bacillus, Staphyloccus, Streptococcus, Enterococcus* and *Clostridium*) of Gram-positive Firmicutes (Fig. 1b; Supplementary Table 1), the most dramatic changes with established CIA occurred within the Clostridiales order. In particular, decreases in the *Clostridiaceae* and the *Lachnospiraceae* (Fig. 1d) families were noted with ES-62 promoting maintenance of the *Ruminococcus, Faecalibacterium* and *Blauti* genera and the family *Erysipelotrichaceae* in addition to the butyrate-producing genera *Dorea* and *Roseburia*, the latter having been implicated in gut health and inflammation homoeostasis^29^. In terms of the Proteobacteria, CIA was associated with outgrowth of members of the *Helicobacter* (Epsilonproteobacteria) and *Escherichia* (Gammaproteobacteria) genera and again this was normalised by ES-62 (Fig. 1e).

Perturbation of the microbiome was also observed in the ileum of CIA mice and again, exposure to ES-62 normalised this towards the healthy community (Fig. 1a). ES-62 clearly promoted growth of the *Clostridiales*, again particularly the *Clostridaceae* and *Lachnospiraceae* families, generally even beyond the levels found in healthy mice (Fig. 1f ). In the context of the relative paucity of bacteria in the ileum relative to the colon, this outgrowth perhaps explains why ES-62 increases the overall species diversity observed in the ileum but not the colon of CIA mice (Supplementary Figure 2). In addition, reflecting that CIA perturbs and ES-62 normalises gut bacteria, functional metagenomic analysis showed that ES-62 generally acted to normalise the metabolic capacity of the colonic microbiome in CIA mice (Fig. 1g). Intriguingly, treatment of Naive, healthy mice with ES-62 for the duration of the CIA model also promotes expansion of members of the *Clostridiales* order, again particularly the *Lachnospiraceae*, whilst decreasing abundance of *Bacilli*, the *Bacteroidaceae* and *Porphyromonadaceae* families of Bacteroidetes and also, Proteobacteria in the ileum (Fig. 1h). Likewise, ES-62 can also promote depletion of Bacteroidetes and expansion of *Ruminococcaceae* and *Clostridiaceae* families and the *Clostridia* class of Firmicutes in the colon of Naive mice (Fig. 1i). Importantly, ES-62’s modulation of the gut microbiota in the absence of the chronic inflammation associated with CIA suggests that it can act directly, and that there is not an absolute requirement for it to harness immunoregulatory mechanisms to maintain and fine-tune microbiome homoeostasis.

### Antibiotics both ameliorate CIA and impact on ES-62 protection

**Fig. 2:**
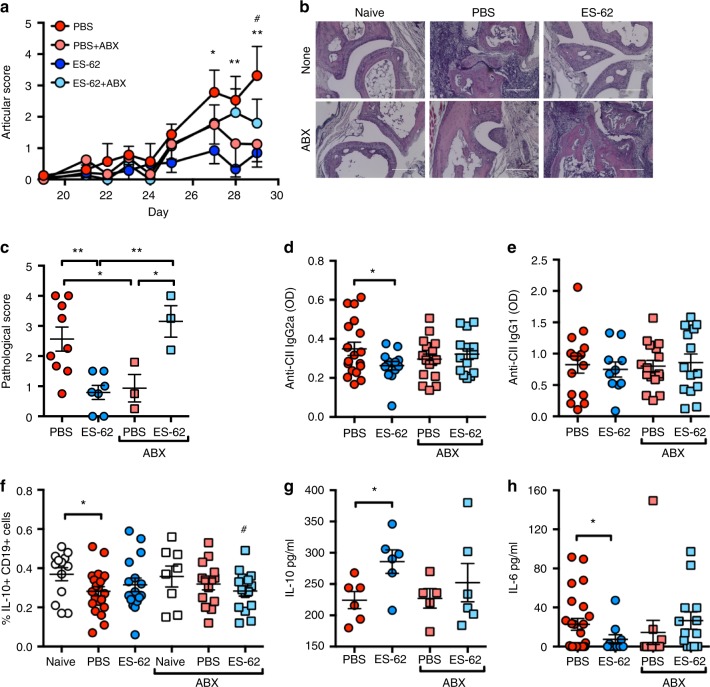
Antibiotic treatment results in an intermediate disease phenotype. **a** Data are presented as articular scores (mean ± SEM) and are pooled from three independent experiments (PBS; *n* = 23, ES-62; *n* = 19, PBS-ABX; *n* = 22 and ES-62-ABX; *n* = 22). **b** H&E staining (scale bars represent 200 µm) of hind paws, from representative mice from each treatment group (clinical articular scores of individual paws were: PBS ≥ 3 (*n* = 9), ES-62 = 0 (*n* = 7), PBS-ABX = 0 (*n* = 3) and ES-62-ABX ≥ 3 (*n* = 3)) selected to compare joint pathology occurring in the similarly scored ES-62-CIA and PBS-ABX (score 0) and ES-62-ABX and PBS-CIA (articular score ≥ 3) mice. **c** Blind scoring of pathology exhibited in joint sections imaged in (**b**). **d**, **e** Levels of collagen II (CII)-specific IgG2a (**d;** PBS; *n* = 18, ES-62; *n* = 13, PBS-ABX; *n* = 16 and ES-62-ABX; *n* = 14) and IgG1 (**e;** PBS; *n* = 15, ES-62; *n* = 10, PBS-ABX; *n* = 16 and ES-62-ABX; *n* = 14) antibodies in serum were determined by ELISA. **f** Splenic B regulatory cells (CD19^+^IL-10^+^ cells) were determined by flow cytometry (Naive; *n* = 13, PBS; *n* = 21, ES-62; *n* = 14, Naive-ABX; *n* = 8 PBS-ABX; *n* = 14 and ES-62-ABX; *n* = 15). **g**, **h** IL-10 (**g**: PBS; *n* = 6, ES-62; *n* = 5, PBS-ABX; *n* = 6 and ES-62-ABX; *n* = 6) and IL-6 (**h**: PBS; *n* = 24, ES-62; *n* = 10, PBS-ABX; *n* = 12 and ES-62-ABX; *n* = 13) concentrations in serum were determined by ELISA. Statistics: all results are presented as mean ± SEM and each symbol represents an individual mouse; data are from one (**g**) or pooled from two or three independent experiments. Statistical significance was determined using two-way ANOVA (**a**) or one-way ANOVA (**c**–**h**) with LSD Fishers multiple comparisons and significance indicated by asterisks, *p < 0.05 and **p < 0.01 and ^#^*p* < 0.05 (PBS vs. PBS + ABX [**a**] and ES-62-ABX vs. Naive [**f**])

To address whether microbiome perturbation observed in CIA plays a role in the initiation and progression of inflammatory arthritis, we investigated the effect of continuous exposure of mice to a cocktail of broad-spectrum antibiotics (ABX) administered from 1 week prior to initiation of CIA. Such ABX treatment had no obvious effect on overall health as, after a characteristic initial dip, there was no significant difference in body weight amongst the CIA groups at cull (Supplementary Figure 2). Nevertheless, this regimen essentially eliminated the bacterial microbiota, irrespective of treatment group, whilst metagenomic analysis showed the residual gut community to be almost entirely comprising proteobacteria (Supplementary Figure 2). As predicted from previous studies in several inflammatory arthritis models^22,30,31^, ABX treatment reduced the incidence (PBS, 65.2%; PBS-ABX, 36.3%, as measured by articular score ≥1) and severity of joint pathology in CIA mice, both in terms of articular score (Fig. 2a) and histopathology (Fig. 2b, c). In addition, the protection afforded by ES-62 was reduced in ABX-treated animals (Fig. 2a). Thus, prophylactic administration of ABX resulted in an intermediate phenotype of CIA, irrespective of whether the mice were treated with PBS or ES-62.

ES-62-mediated protection against CIA is associated with restoration of the homoeostatic balance of regulatory:effector B-cell responses via downregulation of aberrant MyD88 signalling^2,21^. Typically, ES-62 reduces pathogenic anti-collagen type II (CII) IgG2a, but not IgG1 antibody production^32,33^. Reflecting the ABX-driven amelioration of CIA pathology, anti-CII Ig2a levels in PBS-ABX mice are not significantly different from those in ES-62-CIA mice, whilst those in ES-62-ABX mice are not significantly reduced relative to those in PBS-CIA mice (Fig. 2d). No differences were detected amongst any of the groups in terms of anti-CII IgG1 antibodies (Fig. 2e). Consistent with the effects of ABX on effector B-cell responses, analysis of splenic IL-10^+^CD19^+^ Bregs showed that both the decrease occurring during CIA (PBS-CIA) and also the maintenance of healthy levels in ES-62-CIA mice^2,21^ were lost in ABX-treated animals (Fig. 2f). Moreover, whilst ES-62 increases serum IL-10 levels in CIA mice, this regulatory cytokine is found at similarly low levels in PBS-CIA, PBS-ABX and ES-62-ABX mice (Fig. 2g). At the same time, ES-62-mediated suppression of serum levels of IL-6, a cytokine that promotes B-cell (auto)immunity^34^, is lost following ABX treatment (Fig. 2h). Collectively, these ABX studies indicate that depletion of the microbiota interferes with generation of inflammatory mediators associated with CIA pathogenesis as well as with the loss of immunoregulatory elements (Bregs) normally contributing to resolution of chronic inflammation. Importantly, they also indicate that ES-62-resetting of the effector:regulatory balance is dependent on an intact gut microbiome and hence suggest that its restoration is a consequence of ES-62-mediated normalisation of the microbiome dysbiosis associated with CIA.

**Fig. 3:**
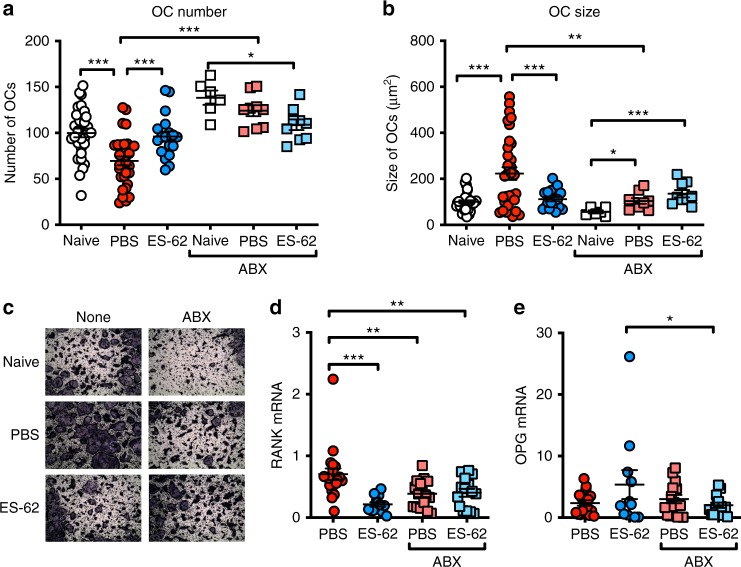
ES-62 requires the gut microbiome to protect the bone remodelling axis. **a**–**c** Osteoclasts (OCs) were differentiated from bone marrow obtained at cull and cultured for 5 days before differentiation, in terms of numbers (**a**) and size (**b**) of OCs, was measured using ImageJ analysis software and data were normalised as a percentage of Naive controls with representative images (**c**) provided (Naive; *n* = 11, PBS; *n* = 11, ES-62; *n* = 6, Naive + ABX; *n* = 2, PBS + ABX; *n* = 3, ES-62 + ABX; *n* = 3). Whole bone marrow was used to quantify RANK (**d**; PBS; *n* = 20, ES-62; *n* = 13, PBS + ABX; *n* = 15, ES-62 + ABX; *n* = 15) and OPG (**e**; PBS; *n* = 17, ES-62; *n* = 11, PBS + ABX; *n* = 16, ES-62 + ABX; *n* = 14) mRNA levels using qRT-PCR, and fold change was calculated following normalisation to Naive controls. Statistics: all data are presented as mean ± SEM. In **a** and **b**, each symbol represents experimental replicates and in **d** and **e** each symbol represents individual mice and data are pooled from three independent experiments. Statistical significance was determined using one-way ANOVA with LSD Fishers multiple comparisons and significance indicated by asterisks, *p < 0.05, **p < 0.01 and ***p < 0.001

ES-62 also acts in CIA mice to suppress the functional maturation of osteoclasts (OC)^20^ that directly cause erosive joint damage. Interestingly, changes in the intestinal microbiome have been shown to impact on bone mass^35,36^ and we therefore investigated whether the intermediate phenotype of joint pathology occurring in ABX-treated CIA mice reflected modulation of osteoclastogenesis resulting from perturbation of the gut microbiota. Exposure of CIA mice to ES-62 restored the numbers of OC differentiated from bone marrow (BM) progenitors ex vivo to Naive levels (Fig. 3a) and blocked their fusion to large, active multinucleated cells (Fig. 3a–c) that resorb bone^20^. Consistent with its amelioration of CIA pathology, ABX administration resulted in a decrease in large multinucleated OCs and a corresponding increase in total numbers of BM-derived OCs from CIA mice (Fig. 3a–c). Under these conditions of microbiota depletion, the ability of ES-62 to inhibit generation of large multinucleated OCs is lost (Fig. 3b): rather, and perhaps consistent with the increased joint inflammation exhibited by these mice, the ES-62-ABX group exhibits the largest multinucleated cells of the ABX-treated cohorts, displaying significantly lower numbers of OCs but larger, multinucleated cells than Naive-ABX mice. ES-62 rewires osteoclastogenesis by modulating the RANK/OPG bone remodelling axis^20^ and this is evidenced again here by its ability to significantly decrease RANK expression and increase (albeit not significantly) expression of the decoy receptor, OPG relative to that seen in BM of PBS-CIA mice (Fig. 3d, e). This axis is also targeted by ABX treatment, with the elevated RANK expression observed in PBS-CIA BM being lost following ABX treatment. In addition, the elevated OPG expression observed in ES-62-conditioned OCs was abrogated following ABX treatment (Fig. 3d, e): these changes would result in similar levels of RANKL-driven osteoclastogenesis consistent with the intermediate CIA phenotype observed in PBS- and ES-62-ABX animals. Supporting these ABX-changes in osteoclastogenesis and consequently bone damage, relative to Naive mice, PBS-ABX and ES-62-ABX animals exhibit similar grip strengths (70.8 ± 5.4% and 58.8 ± 6.0%, respectively) that are intermediate to those displayed by PBS- (42.3 ± 6.2%) and ES-62-CIA mice (90.1 ± 9.6%). Collectively, these data not only support a role for the microbiome in osteoclastogenesis but also implicate its involvement in the ability of ES-62 to modulate pathogenic osteoclastogenesis in CIA.

### ES-62 protects against gut pathology in CIA

**Fig. 4:**
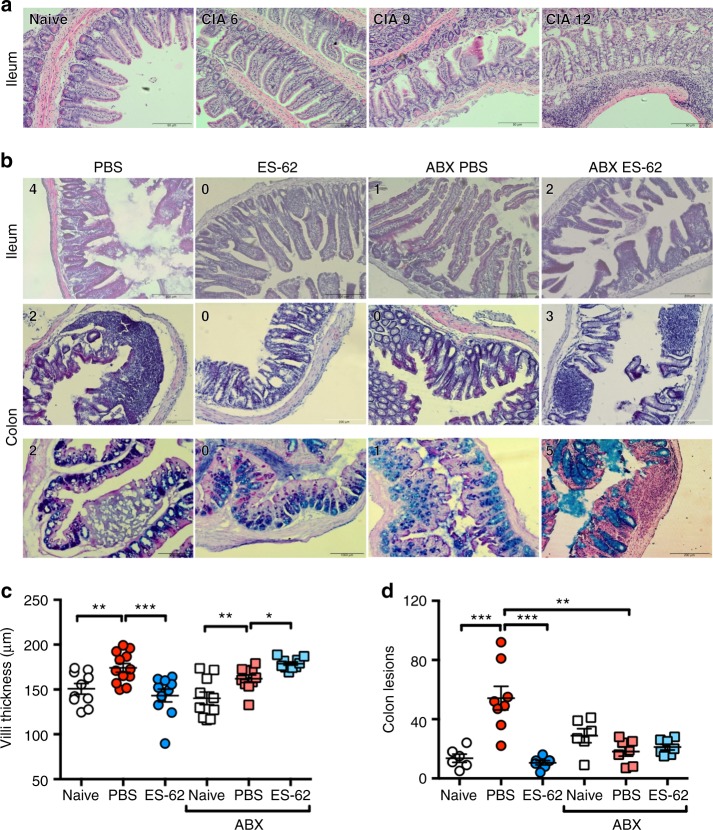
CIA is accompanied by microbiome-dependent gut pathology. **a** Representative H&E images (scale bar representing 50 µm) of ileum sections of Naive and PBS-CIA mice with differential disease severity at cull (mouse articular score as indicated). **b** Representative H&E images of ileum (top row), colon (middle row) and PAS-stained colon (bottom row) sections displaying scale bar (200 µm) and articular scores of the mice are shown on the images. **c** Quantitative analysis of ileum villi thickness, where symbols represent mean values of replicate sections as measured using ImageJ analysis software (*n* = 3/group with 3–5 replicates/animal and data are from two independent experiments). **d** Lesions were enumerated per colon section of individual mice using ImageJ imaging software (Naive; *n* = 6, PBS; *n* = 8, ES-62; *n* = 7, Naive + ABX; *n* = 6, PBS + ABX; *n* = 7, ES-62 + ABX; *n* = 7). Statistics: data are pooled from two independent experiments. Statistical significance was determined using one-way ANOVA with LSD Fishers multiple comparisons and indicated by asterisks, *p < 0.05, **p < 0.01 and ***p < 0.001, whilst in (**c**), PBS-ABX is not significantly different from the Naive group

Intestinal dysbiosis has been associated with loss of gut integrity and chronic inflammation in conditions such as obesity that promote autoimmune disorders like RA and cardiovascular comorbidities^37,38^. Our metagenomic analysis showed that established CIA was associated with gut microbiome changes, in particular with reduction in butyrate-producing bacteria (Fig. 1b, d; Supplementary Table 1) that have been implicated in maintenance of epithelial integrity and gut health^29^. Strikingly, mice with CIA display severe gut pathology and inflammation, the levels of which directly correlate with severity of CIA (Fig. 4a). Moreover, ES-62 treatment protects against such gut damage, specifically the thickening and generation of stubby villi in the ileum (Fig. 4b, c) and hole-like lesions in the colon (Fig. 4b. d). The physiological relevance of these colon lesions is unclear but they may reflect the suppression of mucus production by goblet cells in response to bacteria^39^ or alternatively, attachment/effacement lesions induced by pathogenic bacteria, including *E. coli*^40^. The gut pathology in PBS-CIA mice was diminished in those administered ABX (Fig. 4b–d), suggesting that the associated reduction in chronic gut (and consequently systemic) inflammation contributes to the amelioration of CIA in PBS-ABX mice. Of note, ES-62-ABX mice displayed increased pathology and inflammatory cell infiltration of the gut tissue compared with ES-62-CIA mice (Fig. 4b), findings again consistent with chronic gut inflammation promoting disease severity.

**Fig. 5:**
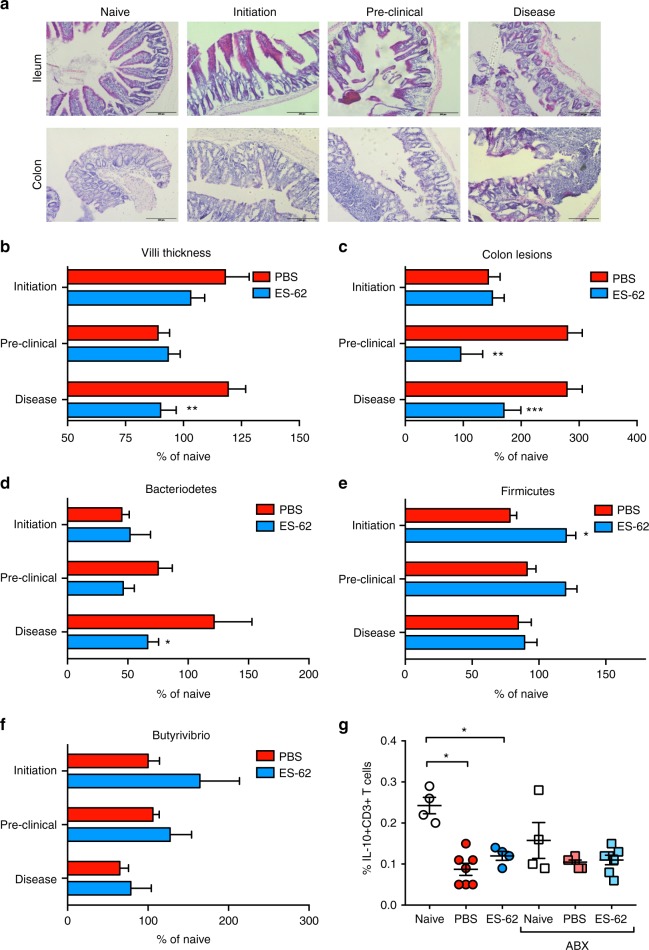
ES-62 protects against gut pathology occurring prior to onset of arthritis. **a** Representative H&E images (scale bars 200 µm) of ileum and colon of mice culled during the Naive, initiation (post immunisation ≤ d14), preclinical (d21 prior to challenge) and disease (established arthritis d ≥ 28, articular score PBS, 5.2 ± 0.8; ES-62–0.9 ± 1.24) phases of CIA. Changes in villus thickness (**b**; Initiation; PBS, *n* = 5, ES-62, *n* = 6, Pre-Clinical; PBS and ES-62, *n* = 3, Disease; PBS, *n* = 4, ES-62, *n* = 5 mice) and number of colon lesions (**c**; Initiation; PBS and ES-62, *n* = 12 images spanning six mice each, Pre-Clinical; PBS, *n* = 6 [3 mice], ES-62, *n* = 4 [2 mice]; Disease; PBS, *n* = 8 [4 mice], ES-62, *n* = 10 [5 mice]) were quantified using ImageJ analysis software with data normalised to values of Naive mice and presented as mean ± SEM. Statistical significance was determined using two-way ANOVA comparing PBS and ES-62 treatment at each time point where **p < 0.01 and ***p < 0.001. **d**–**f** Changes in Bacteroidetes (**d**), Firmicutes (**e**) and Butyrivibrio (**f**) populations in the colon faecal matter of Naive, PBS- and ES-62-treated animals were measured by qPCR, normalised to total bacterial content and presented as % Naive mice. Data are presented as mean ± SEM values from individual animals (Initiation; *n* = 6, Pre-Clinical; *n* = 3, Disease; *n* = 10) and statistical significance determined using two-way ANOVA with LSD Fishers multiple comparisons where *p < 0.05, **p < 0.01 and ***p < 0.001. **g** Tr1 cells (CD3^+^IL-10^+^ cells) were determined by flow cytometric analysis of splenocytes from individual mice (Naive; *n* = 4, PBS; *n* = 7, ES-62; *n* = 4, Naive-ABX; *n* = 4 PBS-ABX; *n* = 6 and ES-62-ABX; *n* = 7) from a single experiment. Statistical significance was determined using one-way ANOVA with LSD Fishers multiple comparisons where *p < 0.05

To further address the role of gut pathology in CIA, we analysed the ileum and colon tissue from Naive mice and those undergoing CIA (treated with PBS or ES-62) at key points during initiation and progression of disease: (i) Naive mice, (ii) the breaking of tolerance and initiation of disease, following immunisation with CII/CFA (≤ day 14), (iii) preclinical (day 21, prior to booster immunisation with CII) and (iv) established disease (≥ day 28; articular score: PBS–5.2 ± 0.8; ES-62–0.9 ± 1.24). This analysis revealed the presence of gut pathology in PBS-CIA mice during the initiation phase with an increase in both ileum villi thickness and colonic lesions following immunisation (Fig. 5a–c). Interestingly, the ileum pathology occurring during the initiation stage in PBS-CIA mice appeared to resolve by the end of the preclinical phase (day 21), although thickening and shortening of the villi was again induced by the booster CII immunisation. This pattern was not the case for the colon lesions, which peaked at day 21 and were maintained throughout active disease in PBS-CIA mice. As opposed to CIA mice, ileum integrity was maintained throughout all phases of disease, whilst the high levels of colon lesions generated by day 21 of the preclinical phase were reduced in ES-62-treated animals (Fig. 5b, c).

Supporting our metagenomics data, qPCR analysis showed that ES-62 prevents the enrichment of Bacteroidetes and maintains levels of Firmicutes in the colon of mice during established CIA (Fig. 5d, e). However, analysis at the various phases of the disease suggested a more dynamic situation: for example, following the primary immunisation with CII/CFA, there is a significant decrease in levels of both Bacteroidetes and Firmicutes, with the rise in Bacteroidetes evident in established arthritis only occurring following the booster immunisation (Fig. 5d, e). By contrast, ES-62 acts to maintain healthy levels of Firmicutes in CIA mice throughout disease, but most strongly during the initiation and preclinical phases and in addition, inhibits the CIA-induced increase in Bacteroidetes during established disease. Moreover, although ES-62 promoted enrichment of butyrate-producing bacteria (*Butyrivibrio*) at all stages of disease, this was most evident in the initiation phase (Fig. 5f). Butyrate has been reported to promote the development of regulatory T cells (both Foxp3^+^ Tregs and IL-10^+^ Tr1 cells^41,42^) which by suppressing and resolving chronic inflammation, could contribute to the maintenance of gut barrier integrity. However, whilst we find that the induction of CIA is accompanied by loss of splenic Tr1 cells, the predominant regulatory T-cell subset defective in CIA^43^, ES-62 does not restore their levels back towards those of Naive mice (Fig. 5g). These data are consistent with our previous findings that protection against CIA afforded by ES-62 is not associated with induction of either Treg or Tr1 cell responses^21^. Moreover, ABX treatment does not modulate either the CIA-associated depletion of Tr1 cells or the failure of ES-62 to induce their restoration, suggesting their development in this model is not impacted by the status of the microbiome (Fig. 5g). Thus, collectively these data indicate that ES-62 likely promotes *Butyrivibrio* species to maintain butyrate-mediated protection of intestinal epithelium physiology and barrier integrity^44^.

**Fig. 6:**
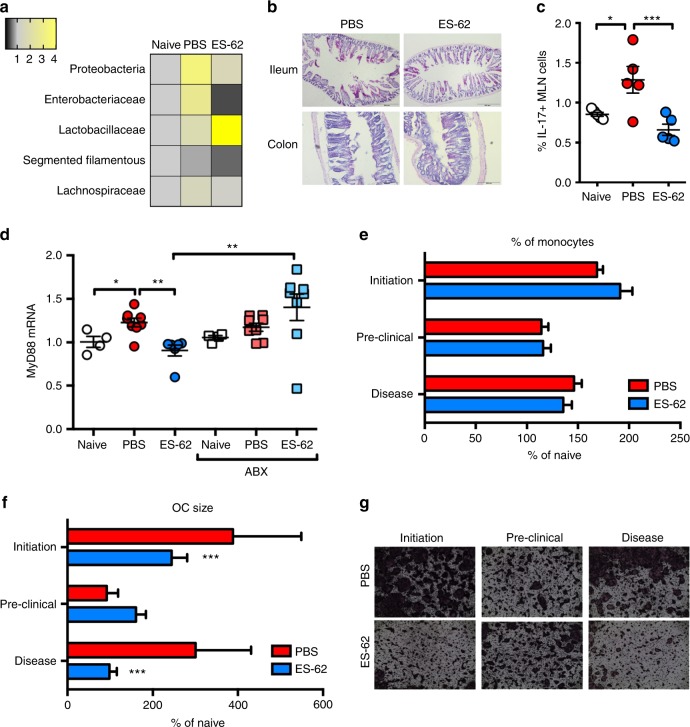
ES-62 modulates the gut-bone marrow axis during the early phases of CIA. **a** Changes in the indicated bacterial populations in the colon faecal matter of Naive, PBS- or ES-62-treated animals were measured at day 6 post CII/CFA-immunisation by qPCR and data were normalised to total bacterial content and presented as fold change compared to Naive mice (Naive, *n* = 3; PBS-CIA, *n* = 3 and ES-62, *n* = 2). **b** Representative H&E images of ileum and colon pathology of mice culled at day 6 of CIA. Scale bars are 200 µm. **c** IL-17^+^ lymphocytes were determined by flow cytometric analysis of MLN cells from individual mice (naïve, *n* = 5: PBS, *n* = 5 and ES-62, *n* = 5). Statistical significance was determined using one-way ANOVA with LSD Fishers multiple comparisons and significance indicated by asterisks, *p < 0.05 and ***p < 0.001. **d** Whole bone marrow was used to quantify MyD88 mRNA levels using qRT-PCR and normalised to naive controls. **e** The proportion of monocytes (CD3^-^B220^-^Ter119^−^Ly6G^−^Ly6C^+^) in bone marrow was measured by flow cytometry and normalised to those in Naive control mice. **f**, **g** Osteoclasts (OCs) were differentiated from bone marrow obtained at cull and cultured for 5 days and size of OCs (**f**) was measured using ImageJ analysis software and normalised to those from Naive controls with representative images for each disease stage in both treatment groups displayed (**g**). Statistics: data are presented as mean ± SEM values from individual animals (**d;** Naive; *n* = 4, PBS; *n* = 8, ES-62; *n* = 6, Naive + ABX; *n* = 4, PBS + ABX; *n* = 8, ES-62 + ABX; *n* = 8. **e** Initiation; *n* = 6, Pre-Clinical; *n* = 3, Disease; *n* = 4) or mean ± SD from experimental replicates (**f**; Initiation; *n* = 18, Pre-Clinical; *n* = 9, Disease; PBS: *n* = 12 and ES-62; *n* = 15). Statistical significance was determined using two- (**e**, **f**) or one-way (**d**) ANOVA with LSD Fishers multiple comparisons and indicated by asterisks, *p < 0.05, **p < 0.01 and ***p < 0.001

The dynamic changes observed in the gut during the initiation phase further questions whether CIA-associated autoimmune (Th17) inflammation is responsible for triggering gut pathology and consequently, perturbation of the microbiome or vice versa. Interestingly, therefore, whilst confirming our findings that dysbiosis and loss of intestinal barrier integrity are established by day 14 in the CIA model, a very recent report^45^ shows that such breakdown in gut function precedes, and is required for, CIA-associated IL-17 responses as increases in IL-17 can only be determined in the small intestine from day 14 whilst those in the MLN occurred even later, being present during the established disease phase (by day 35, but not detected on day 14), and that ABX-depletion of the microbiota inhibits such IL-17 production and severity of joint disease. Moreover, these effects appeared not to require (any) inflammation for initiation as they could not be replicated by sham immunisation with CFA^45^. Consistent with this idea that dysbiosis and associated gut pathology precedes and shapes the autoimmune inflammation driving arthritis, we find that CIA-associated perturbation of the colon microbiota is evident as early as day 6 (post CII immunisation): this is broadly normalised by exposure to ES-62, with the worm product modulating levels of potentially pathogenic bacteria (such as the *Enterobacteriaceae* family of Gamma proteobacteria) to below those found in Naive animals (Fig. 6a). Accompanying the CIA-dysbiosis, there is damage to both the ileum and colon, with shortening and thickening of the ileum villi particularly evident at this early time point and this is protected against by ES-62 (Fig. 6b). These changes in gut function occur in the absence of the systemic inflammation (serum IL-6 cytokine and CII-specific IgG2a antibodies were only present around the limit of detection of the ELISAs employed, with no differences observed amongst Naive, PBS-CIA and ES-62-CIA groups), known to be targeted by ES-62 in CIA (Fig. 2). By contrast, the late microbiota-driven IL-17 responses developed in the MLN in the clinical phase of CIA^45^ were suppressed by exposure to ES-62 (Fig. 6c). Collectively, these findings indicate that ES-62 primarily acts directly to normalise the CIA-associated gut dysbiosis that both triggers mucosal inflammation and initiates the mesenteric lymph node (MLN) IL-17 responses that drive autoimmunity, rather than indirectly modulating the dysbiosis and gut pathology arising from the systemic inflammation that results from the CIA-immunisation protocol.

Although the precise details of how gut microbiome perturbation occurs and drives chronic autoimmune inflammation and joint destruction in CIA remain to be defined, commensal bacteria play key roles in educating the immune system and BM progenitors and hence, loss of microbiome diversity can impact on both inflammation and bone homoeostasis^46,47^. Such training involves interactions of Pattern Recognition Receptors (PRR; e.g., TLRs and NODs) with the gut microbiome^2,3,48^. Consistent with this, ES-62 can rewire BM progenitors and stromal cells from CIA mice to an anti-inflammatory, regulatory or tissue repair phenotype^2,16,20,49–51^ by subverting TLR4 signalling to prevent the upregulation of the key TLR signal transducer, MyD88 observed during chronic inflammation^52^. Interestingly, therefore, given the increased incidence and severity of CIA in ES-62-ABX mice, we show that ES-62 dampening of aberrant MyD88 expression is abolished by ABX treatment (Fig. 6d).

Commensal bacteria can also dynamically educate OC progenitor (OCP) maturation^46,47^: as OCs normally act in concert with osteoblasts (OBs) to homeostatically maintain healthy bone, the enhanced functional capacity of OCs elicited by commensal bacteria may actually render hosts more susceptible to bone damage during chronic inflammation, a condition that promotes bone remodelling, particularly as commensal bacteria also appear to act to suppress OB function^46,47^. Reflecting the dynamic and differential changes in the colonic microbiota, gut inflammation and pathology, there is a strong increase in the monocyte populations containing OCPs during the early inflammatory initiation phase of CIA that has resolved by the end of the preclinical phase only to increase again in the established phase of arthritis following the booster CII immunisation. Consistent with our data that ES-62 does not fundamentally suppress OC differentiation, there are no differences in the percentage of monocytes between the PBS-CIA and ES-62-CIA groups (Fig. 6e). However, following both primary and booster immunisations, BM from CIA mice shows enhanced capacity for functional OC maturation that is suppressed by ES-62 (Fig. 6f, g).

## Discussion

This study demonstrates that a defined parasitic worm-derived product can impact on, and harness, the microbiome to exert its therapeutic effects against chronic inflammation in target organs distal to the gut, such as the joints. Collectively, our data suggest that ES-62-mediated protection reflects broad normalisation of the gut dysbiosis observed in arthritic mice.

Potentially pathogenic changes in the gut microbiome have been described following analysis of stool faeces from patients with new-onset disease (enrichment of *Prevotella copri*^10,53^), as well as those with established arthritis (changes in the *Clostridiaceae, Coriobacteriaceae* and *Lachnospiraceae* families^54,55^). Perhaps reflecting this, our metagenomic analysis of CIA mice during the established disease phase shows ES-62’s protective actions to be most strongly associated with maintenance of the *Clostridiaceae, Lachnospiraceae and Ruminococcaceae* families, particularly those associated with butyrate production, e.g., *Ruminococcus*. Administration of butyrate was previously found to ameliorate CIA severity, notably in terms of reduction in each of inflammatory cell infiltration of the joint, pannus formation and cartilage and bone destruction^56^. By contrast, butyrate exacerbated antibody-induced arthritis, a model which bypasses the initiation and adaptive immunity phases of disease, suggesting that butyrate needs to act during these preclinical phases to exhibit its protective actions^56^. Possibly consistent with this, whilst ES-62 protects against depletion of butyrate-producing species in mice with established disease, we also find *Butyrivibrio* to be most enriched by ES-62 in the CIA initiation phase. Collectively, these data suggest that depletion of butyrate-producing bacteria associated with onset of CIA may contribute to the gut pathology promoting and perpetuating the inflammation that drives immune tolerance breakdown and consequent autoimmune joint disease. Certainly, butyrate is known to regulate gut barrier integrity^44^ and goblet cell production of MUC2^57^. Indeed, we find microbiome dysbiosis and gut pathology to precede joint disease onset, being observed within 6 days of primary CII immunisation and in apparent absence of systemic inflammation. Furthermore, the loss of colon barrier integrity peaks by the end of the CIA preclinical phase (d21) and such gut pathology is accompanied by dynamic changes in the microbiome of CIA mice as evidenced by the decrease in colon abundance of Bacteroidetes and Firmicutes during the early initiation phase of disease and the enrichment of the former during established arthritis.

The normalisation of the gut microbiota by ES-62 may actually result in a dual pronged mechanism by which butyrate, in addition to its local gut-protecting actions, could also impact systemically to protect more directly against joint pathology. Consistent with this, butyrate has been reported to suppress osteoclastogenesis^58^, and by protecting against pathological bone loss, to regulate bone mass^35^. Intriguingly, we find spikes of functional maturation of OCs in the initiation and established phases of CIA that are prevented by ES-62 and are associated with its enrichment of *Butyrivibrio*. The mechanisms by which ES-62 orchestrates such maintenance of the complex homoeostasis of the gut-bone marrow axis are not clear. However, as ES-62 can modulate the gut microbiota in Naive healthy mice, it appears that such fine-tuning of the microbiome can occur directly, rather than indirectly via potential anti-inflammatory and immunoregulatory actions that suppress gut pathology, to protect against dysbiosis. It is therefore possible that ES-62 may act in a manner analogous to quorum-sensing molecules^59,60^, which are produced by bacteria and signal to regulate population densities within a particular microbial niche. Thus, by coordinating gene expression responses both within and across species, quorum sensing molecules shape the microbial community and its interaction with the host, particularly with respect to virulence and pathogenesis;^59,60^ our future plans therefore encompass determining whether ES-62 can modulate bacterial growth directly as a starting point in investigating whether quorum-sensing activities contribute to its ability to normalise microbiome dysbiosis and maintain gut homoeostasis.

However, in terms of CIA, our current working model is that the induced dysbiosis may impact on MyD88-integrated microbiota-sensing intestinal epithelial-dendritic cell interactions^61,62^ to drive gut pathology and loss of barrier integrity and to train inflammation and (auto)immune responses that in turn impact on the microbiome and perpetuate chronic pathology. ES-62 exploits TLR4 to sense (aberrant) MyD88 signalling^2^ allowing such changes in the microbiome to be detected by the helminth product and enabling it to dynamically stabilise intestinal epithelial barrier function and integrity and hence fine-tune bacterial species diversity and abundance to homeostatically maintain a healthy microbiome. It is interesting therefore in this context that systemic Th17 differentiation and consequent autoimmune arthritis occurring in the IL1rn^−/−^ model of RA is dependent on TLR4^9,30^. Moreover, the accompanying dysbiosis, that on faecal transfer can confer arthritis-predisposing Th17 inflammation in wild-type mice, is also regulated by TLR4^30^. Furthermore, interestingly, 11/44 taxa disrupted in IL1rn^−/−^ mice were normalised in IL1rn^−/−^TLR4^−/−^ animals and these included *Ruminococcus* species, which are also promoted by ES-62^30^.

In mechanistic terms, Breg levels—reset by ES-62 in CIA—have also been reported to be regulated by the microbiome in other models of autoimmune arthritis^22^. Indeed, consistent with the proposal that Bregs are homeostatically induced to resolve dysbiosis-induced inflammation in autoimmune arthritis, as indicated by disruption of the process by ABX treatment^22^, we have similarly found the ES-62-mediated restoration of IL-10^+^ B cells, as well as the suppression of pathogenic anti-CII antibody and IL-6 production, in CIA to be compromised by such perturbation of the microbiome. Collectively, these findings suggest that by targeting MyD88 to maintain microbiome homoeostasis, ES-62 may subsequently achieve its immunoregulatory effects, which in turn, by dampening and resolving chronic inflammation, further contribute to the normalisation of intestinal barrier integrity and the gut microbiome.

Given that normalisation of the gut microbiome appears central to ES-62 resetting of immunoregulation in CIA, at first sight it may appear rather counter-intuitive that the worm product fails to afford protection^63^ in mouse models of type-1 diabetes (T1D), multiple sclerosis (MS) and inflammatory bowel disease (IBD); conditions which have each been reported to exhibit patterns of microbiome dysbiosis. However, it is likely that at least in the dextran sodium sulphate (DSS) model of IBD that the severe physical damage to the intestinal epithelial barrier bypasses the capacity of ES-62 to normalise the microbiome, as the resultant leakage and aberrant bacterial colonisation would generate inflammation and pathology. In addition, loss of regulatory T-cell function appears necessary for pathogenesis in each of the T1D, MS and IBD models tested and ES-62 appears to lack the capacity to restore either Treg or Tr1 responses in all of the models of inflammatory diseases we have tested to date^2,63^. This is perhaps rather surprising given the well-documented induction of regulatory T cells by helminths^2^ and the capacity of T2-MZP Bregs, to induce Tr1 and Tregs^64^. However, we do not see significant induction of T2-MZP Bregs, but rather a general upregulation of IL-10^+^ B cells in the CIA model^21^ where interestingly, we also fail to detect any impact of the microbiome on Tr1 responses. Thus, one possibility is that ES-62 only protects in models where (microbiota-regulated) Bregs can directly mediate immunoregulatory effects, such as CIA, MRL/Lpr-SLE and asthma models^2^. Consistent with this, whilst roles for Bregs have been proposed in MS and IBD (DSS) models, these appear to have been dependent on interactions with regulatory T cells and indeed, the IBD-T cell transfer models tested were performed in Rag1^−/−^ mice that cannot develop B cells^63^.

Finally, TLR4/MyD88 signalling in RA had previously been attributed solely to recognition of DAMPs in the joint^65^ and thus collectively, our findings shed new light on its pathogenic roles in initiation and progression of disease as well as emphasise its potential as a therapeutic target in RA. In particular, they underscore the complex and central role of TLR4/MyD88 signalling in regulating the gut-bone marrow axis in musculoskeletal homoeostasis and its dysregulation resulting in systemic inflammation, breaking of tolerance, aberrant osteoclastogenesis and consequently joint destruction in arthritis. Moreover, they suggest that ES-62 may achieve its protective effects in CIA by directly targeting this key regulatory node to normalise microbiome dysbiosis and associated gut pathology in order to rebalance the gut-bone marrow axis and limit aberrant inflammation and joint damage, by consequently homeostatically restoring levels of Bregs and resetting osteoclastogenesis. Thus, by exploiting ES-62 as a unique tool to dissect pathogenic and protective microbial signatures in CIA, we could potentially understand how to elicit homoeostatic regulation of the gut and resolve inflammation in autoimmune inflammatory arthritis.

## Methods

### Collagen-induced arthritis

Male DBA/1 mice were purchased at 6–8 weeks of age (Envigo; Bicester, UK) and housed and maintained in the Central Research Facility of the University of Glasgow. All experiments were approved by, and conducted in accordance with, the Animal Welfare and Ethical Review Board of the University of Glasgow, UK Home Office Regulations and Licences PPL P8C60C865, PIL I518666F7, PIL 1675F0C46 and PIL ICEBDB864.

CIA was induced using bovine Collagen Type II (CII: 100 μg) emulsified with complete Freund’s adjuvant (MD Biosciences) injected intradermally on day 0. Mice were challenged with 200 μg of CII in PBS intraperitoneally on day 21. Animals were treated with PBS or purified endotoxin-free ES-62 (2 µg/injection) subcutaneously on days −2, 0 and 21, and joint inflammation and damage (articular score) were determined as described previously^16,32,50^. Grip strength was recorded as per the manufacturer’s instruction (Ugo Basile®, Italy) using a Gripometer, which measured the grip strength (peak force and time resistance) of the forelimbs of the mice. The animals were placed over a base plate and gripped a T-shaped grasping bar, which was connected to the peak amplifier that automatically detects the animal’s response. Three measurements were taken and the average grip strength was calculated. In order to investigate the impact of gut microbiome perturbation on initiation and progression of inflammatory arthritis, animals were given drinking water containing [or not] a cocktail of antibiotics (500 mg/L Vancomycin, 1 g/L Neomycin and 1 g/L Metronidazole) to eliminate Gram-positive, Gram-negative and anaerobic microorganisms^22^ 7 days prior to the induction of CIA and thereafter continuously throughout the experiment. Blood was sampled using endotoxin-free needles, and syringes and the resulting serum isolated and stored at −20 °C in endotoxin-free Eppendorf tubes. Paw, ileum and colon tissue was fixed in 4% paraformaldehyde; ileum and colon faecal contents were collected in sterile RNAlater (Sigma, UK) and stored at −80 °C. Endotoxin-free ES-62 was purified from spent culture medium as described in detail previously^32^.

### Flow cytometry

Spleen, lymph node (LN) and bone marrow (BM) cells were suspended in FACS buffer (2.5% BSA; 0.5 mM EDTA, in PBS) following red blood cell lysis (eBioscience, UK). BM cells were labelled with a cocktail of PE-labelled antibodies specific for CD3 (catalogue number: 100205), B220 (catalogue number: 103207) and Ter119 (catalogue number: 116207) to exclude analysis of lymphocytes and erythroid cell populations using a dump channel, and monocytes were identified by labelling with antibodies against CD11b (FITC; catalogue number: 101206), Ly6C (PerCP Cy5.5; catalogue number: 128011) and Ly6G (APC; catalogue number: 127613)^20^. Lymphocytes were labelled with antibodies specific for CD3 (FITC; catalogue number: 100305/6), CD19 (AF700; catalogue number: 115527/8), IL-10 (PE or APC; catalogue number: 505007/9) and IL-17 (PerCP5.5 or APC; catalogue number: 506915/6/9/20). For surface marker staining, antibodies were used at 0.2 µg/10^6^ cells (1/100 dilution) except for anti-CD45, which was used at 1/200 dilution. For Fix/Perm staining of intracellular cytokines, 1 µg/10^6^ cells (1/20 dilution) were used. Gating strategies are shown in Supplementary Figure 3. All antibodies were purchased from BioLegend, UK. Fixable viability stain (APC-ef780; ThermoFisher Scientific, UK) was used to select for live cells and for analysis of IL-10^+^ regulatory B cells (Bregs), lymphocytes were stimulated with PMA, ionomycin, Brefeldin A and LPS as described previously^18,21^. Data were acquired using a FACS Canto or BD LSRII flow cytometer and populations were gated as described previously using isotype and fluorescence minus one (FMO) controls using FlowJo, LLC analysis software (Tree Star/BD)^18,20,21,50,66^.

### Histology

Ileum, colon and joint (paw) tissues from individual mice in each treatment group were fixed in 4% paraformaldehyde for 24 h before gut tissues were embedded in OCT and paw joints were decalcified and subsequently paraffin embedded. Paraffin sections (6 μm) and OCT cryosections (9–10 μm) were prepared and standard H&E histological staining was performed on all tissues for identification of morphological changes^16,20,50^. Ileum (villi thickness) and colon (number of lesions) pathology was quantitated by ImageJ analysis. Joint pathology was scored according to the grading system of 0 for no inflammation, 1 for mild inflammation, pannus formation and bone damage, up to a score of 4 representing a high level of inflammation, pannus infiltration and bone and cartilage destruction, as previously described^67^.

### Osteoclast differentiation

OCs were differentiated from BM obtained from the hind limbs of experimental animals as previously described^20^. Briefly, following removal of adherent cells, BM cells were cultured in αMEM medium supplemented with 30 ng/ml M-CSF and 50 ng/ml RANKL (Peprotech, London, UK) and then assessed for OC differentiation by TRAP staining (Leukocyte Acid Phosphatase Kit, Sigma-Aldrich, UK) on day 5. Images were obtained using an EVOS FL Auto Cell Imaging System. TRAP^+^ cells with ≥ 3 nuclei were enumerated, and ImageJ software was used to calculate the average size of multinucleated OCs per field of view (FoV)^20^.

### Serum cytokine and antibody ELISAs

Interleukin-6 (IL-6) and IL-10 expression was measured by ELISA according to the manufacturer’s instructions (BD Biosciences, Oxford, UK). For determination of collagen type II (CII)-specific IgG1 and IgG2a antibodies in serum^32^, high-binding 96-well ELISA plates were coated with CII (5 µg/ml) overnight at 4 °C before washing and blocking with BSA/PBS. Serum was diluted 1:100 and then serially diluted threefold until 1:218,700 and incubated with HRP-conjugated goat anti-mouse IgG1 or IgG2a (1:10,000) in 10% FBS/PBS prior to developing with TMB and 2 M sulphuric acid and read at an optical density of 450 nm.

### qRT-PCR

BM cells (10^6^) were lysed in RLT lysis buffer prior to mRNA extraction using RNeasy Plus Mini kit (Qiagen, Germany) according to the manufacturer’s instructions. The High Capacity cDNA Reverse Transcriptase kit (Applied Biosystems, Life Technology, UK) was used to generate cDNA for use with StepOne Plus™ real-time PCR system (Applied Biosystems, UK) and KiCqStart® qPCR Ready Mix (Sigma-Aldrich). Pre-designed KiCqStart™ primers (Sigma-Aldrich) were purchased to evaluate RANK (*tnfrsf11a*; forward: GAAATAAGGAGTCCTCAGGG, reverse: GAAATAAGGAGTCCTCAGGG), OPG (*tnfrsf11b*; forward: GAAGATCATCCAAGACATTGAC, reverse: TCCTCCATAAACTGAGTAGC), MyD88 (*myd88*; forward: GAAGATCATCCAAGACATTGAC, reverse: TCCTCCATAAACTGAGTAGC) and β-actin (*actb*; forward: GATGTATGAAGGCTTTGGTC, reverse: TGTGCACTTTTATTGGTCTC). Data were normalised to the reference gene β-actin to obtain the ΔCT values that were used to calculate the fold change from the ΔΔCT values following normalisation to biological control group.

### Metagenomics

Given the well-documented variation amongst individual animals that typically exhibit differential scores/affected joints in the CIA model and the range of environmental parameters impacting on the microbiome^68,69^, we attempted to minimise variation by pooling samples from three mice of similar disease score/group from each independent CIA model rather than analysing each individual mouse. Furthermore, pooling of samples also reduces variation in sample and library preparation, whilst barcoding of these pooled samples allowed analysis of the different treatment groups on a single chip and provided internal controls for comparison of treatment groups within each experiment. This process was repeated for three independent CIA models (involving nine representative naive, PBS-CIA [articular score: 7.00 ± 0.91] and ES-62-CIA [articular score: 0.50 ± 0.33, where ***p < 0.001 for the PBS-CIA versus naive and ES-62-CIA cohorts] mice overall), further reducing the impact of disease score and individual variation.

Genomic DNA from the ileum and colon faecal matter was purified using QIAamp DNA Stool Mini Kit (Qiagen, Germany) and stored at −20 °C. For shotgun metagenomic analysis using the Ion Torrent PGM™ platform, samples from three individual mice per group were pooled and between 10 and 100 ng of the pooled DNA was fragmented (NEB Fast DNA Fragmentation & Library Prep Set for Ion Torrent, NEB Inc, UK) and barcoded (IonXpress Barcode Adapters Kit, ThermoFisher Scientific, UK). Barcoded libraries were quantified using a Qubit Fluorometer (ThermoFisher Scientific, UK) and bioanalyser (High Sensitivity DNA analysis Kit, Agilent, UK). Up to three barcoded libraries were combined per Ion 316™ Chip Kit v2 following library preparation using the Ion PGM™ Hi-Q™ View OT2 and Ion PGM™ Hi-Q™ View Sequencing Kits (ThermoFisher Scientific, UK). Data were extracted as FASTQ files and analysed using MG-RAST to generate taxonomic data from sequencing reads^70^. The number of reads per phylum, class, order, family, genera or species of interest were expressed as a composition of all bacteria present to normalise for variation between sequencing runs. Sequencing runs can be accessed using MG-RAST IDs; mgm4777616.3, 4777615.3, 4777614.3, 4777613.3, 4777481.3, 4777480.3, 4777479.3, 4777478.3, 4767994.3, 4767993.3, 4767992.3, 4767991.3, 4767990.3, 4767989.3, 4767988.3, 4767987.3, 4767986.3, 4738191.3, 4738190.3, 4738025.3, 4737887.3, 4737053.3 and 4737052.3. qPCR was used to validate changes in bacterial populations using primers specific for Bacteroidetes (forward: GTTTAATTCGATGATACGCGAG, reverse: TTAAGCCGACACCTCACGG)^71^, Firmicutes (forward: GGAGCATGTGGTTTAATTCGAAGCA, reverse: AGCTGACGACAACCATGCAC)^71^, *Butyrivibrio* (forward: GCGAAGAAGTATTTCGGTAT, reverse: CCAACACCTAGTATTCATC)^72^, Proteobacteria (forward: TCGTCAGCTCGTGTCGTGA, reverse: CGTAAGGGCCATGATG)^73^, Enterobacteriaceae (forward: GTGCCAGCAGC CGCGGTAA, reverse: GCCTCAAGGGCACAACCTCCAAG)^74^, Lactobacillaceae (forward: TGGAAACAGGTGCTAATACCG, reverse: GTCCATTGTGGAAGATTCCC)^75^, segmented filamentous bacteria (forward: GACGCTGAGGCATGAGAGCAT, reverse: GACGGCACGG ATTGTTATTCA)^76^, Lachnospiraceae (forward: CGGTACCTGACTAAGAAGC, reverse: AGTTTCATTCTTGCGAACG)^77^ and were normalised to the total levels of bacteria using pan-bacterial primers (forward: CGGTGAATACGTTCCCGG, reverse: TACGGCTACCTTGTTACGACTT)^77^.

### Statistics

All data were analysed using GraphPad Prism 6 software using one- or two-way ANOVA with Fishers LSD post-tests for parametric data or Kruskal–Wallis test and Dunn’s post test for non-parametric data. Unsupervised hierarchical clustering with Euclidean distance was performed on the colon samples using the heatmap.2 function of the gplots package in R. Supervised heatmaps were generated using GraphPad Prism 7 software. Indicators of significance include *p < 0.05, **p < 0.01 and ***p < 0.001.

### Reporting Summary

Further information on experimental design is available in the Nature Research Reporting Summary linked to this article.

## Supplementary information

Supplementary Information

Reporting Summary

## Data Availability

The data supporting the findings are all contained within the article and Supplementary Information files. In addition, the metagenomic data are available from the MG-RAST database using the accession numbers provided in the Methods section. Other primary data files are available from the corresponding authors on reasonable request.
