# Supplementary material for: The parasitic worm product ES-62 normalises the gut microbiota bone marrow axis in inflammatory arthritis
Source: Nat Commun. 2019 Apr 5;10:1554. doi: 10.1038/s41467-019-09361-0 (PMC6451002; doi:10.1038/s41467-019-09361-0)
Supplement: Supplementary file 1 — Supplementary Information [file 41467_2019_9361_MOESM1_ESM.pdf]

The parasitic worm product ES-62 normalises the gut microbiota bone marrow axis in inflammatory arthritis

Doonan et al.

Supplementary Figures

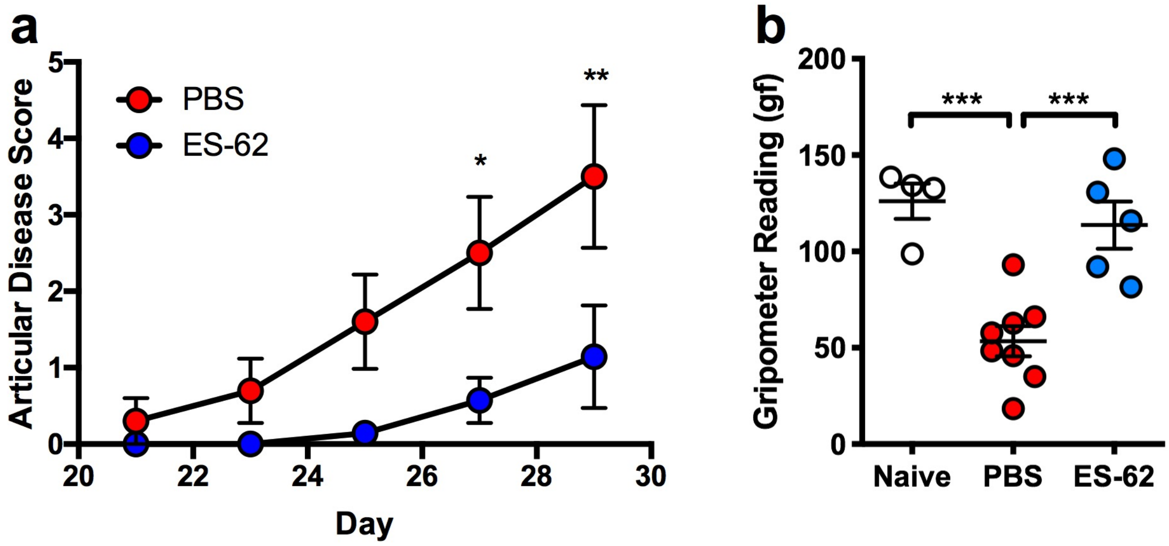

Supplementary Fig. 1. ES-62 protects against CIA (**a**) Articular scores expressed as means ± SEM of PBS- (n = 10) or ES-62- (n = 7) treated CIA animals, with data from a single experiment. (**b**) Grip strength (gf) expressed as an average of three measurements per mouse determined prior to cull as an indicator of forelimb strength and joint morbidity (Naive, n = 4; PBS, n = 8 and ES-62, n = 6). Statistical significance was determined using two-way (**a**) and one-way ANOVA (**b**) with LSD Fishers multiple comparisons and for levels of significance where indicated by asterisks, * = $p < 0.05$, ** = $p < 0.01$ and *** = $p < 0.001$.

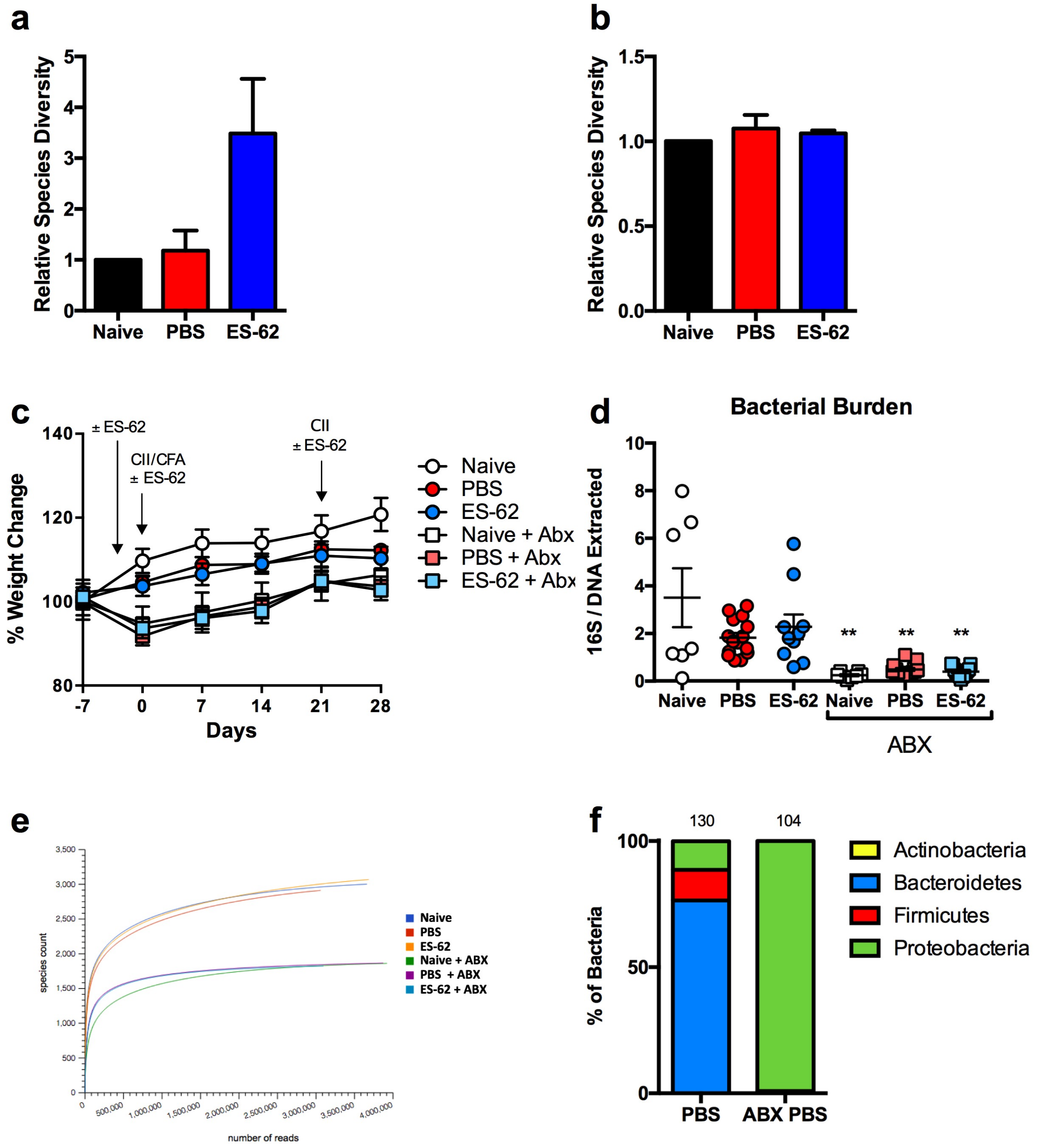

Supplementary Fig. 2. Species diversity in CIA and depletion by antibiotics (ABX). The number of species detected during metagenomic analysis of the ileum (**a**) and colon (**b**) of Naive, PBS-CIA and ES-62-CIA mice. Data are presented as mean ± SEM where for metagenomic analysis, three mice per group were pooled from three independent experiments and normalised to Naïve controls. (**c**) Body weights of individual animals over the time course of CIA were measured from day -7 (prior to ABX administration)

and presented as mean % weight ± SEM (Naïve; n = 8, PBS; n = 16, ES-62; n = 13, Naive+ABX; n = 8, PBS+ABX; n = 16, ES-62+ABX; n = 15). (**d**) DNA was isolated from the colon contents of individual animals and the levels of 16S bacterial DNA detected by qPCR was measured and normalised to the levels of total DNA extracted. Symbols represent values from individual mice from a single experiment. (**e**) Representative rarefaction plots from the MG-RAST metagenomic analysis showing the levels of bacterial diversity of colon faecal matter in each treatment group from one of three experiments. (**f**) Composition of bacterial phyla present in PBS or PBS-ABX animals (metagenomic data obtained by pooling samples from 3 mice/group from a single experiment) represented as a % of all bacteria detected. Statistics: data are presented as mean ± SEM (**c** and **d**). Statistical significance was determined using one-way ANOVA with Dunn's multiple comparisons comparing Naïve, PBS or ES-62-treated groups to their respective ABX-treated control and indicated by asterisks ** = $p < 0.01$.

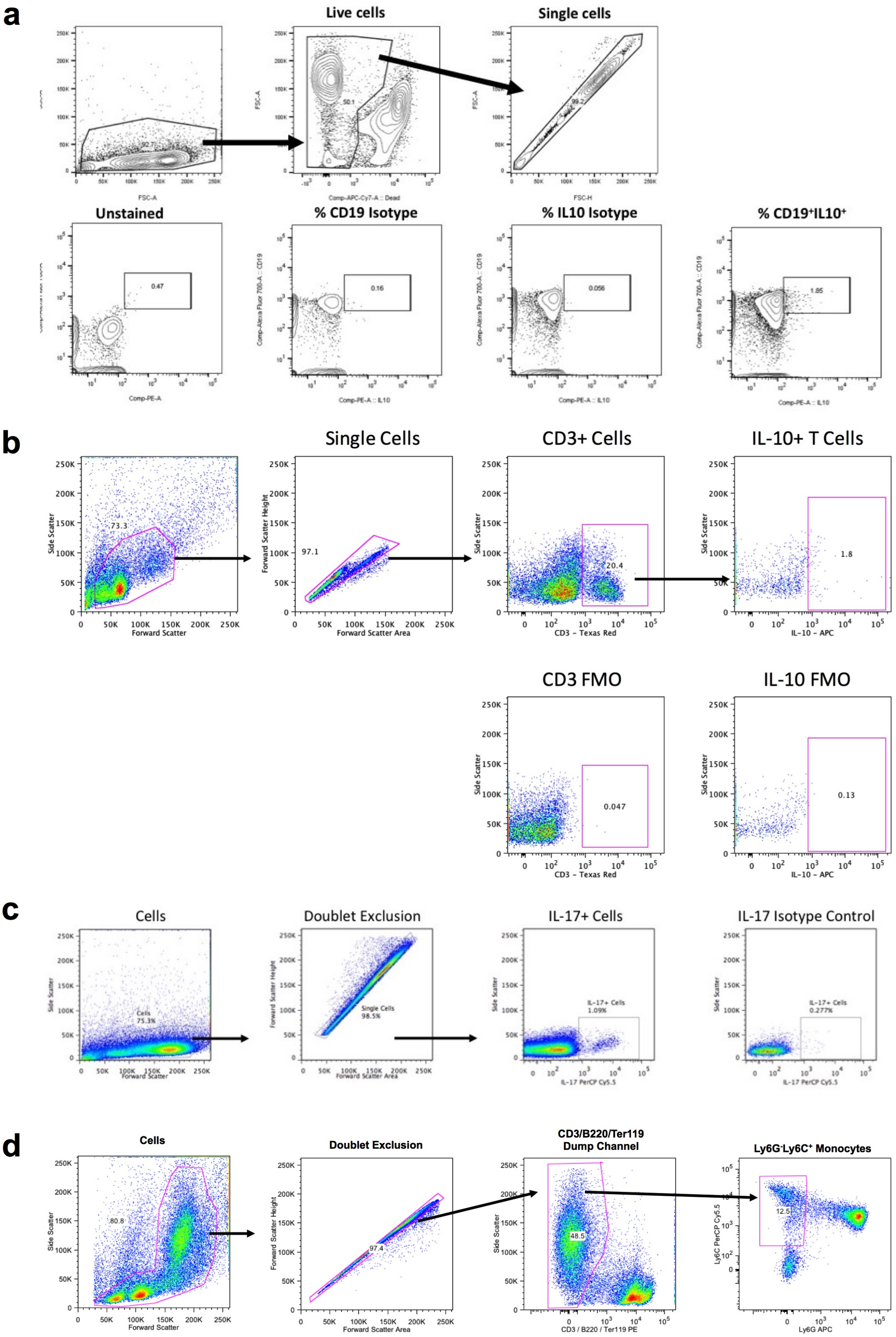
a
Live cells
Single cells
92.7
50.1
99.2
FSC-A
FSC-H
Comp-APC-Cy7-A :: Dead
Unstained
% CD19 Isotype
% IL10 Isotype
% CD19+IL10+
0.47
0.16
0.056
1.85
Comp-PE-A
Comp-PE-A :: IL10
Comp-Alexa Fluor 700-A :: CD19
b
Single Cells
CD3+ Cells
IL-10+ T Cells
73.3
97.1
20.4
1.8
Side Scatter
Forward Scatter
Forward Scatter Height
Forward Scatter Area
CD3 - Texas Red
IL-10 - APC
CD3 FMO
IL-10 FMO
0.047
0.13
c
Cells
Doublet Exclusion
IL-17+ Cells
IL-17 Isotype Control
Cells 75.3%
Single Cells 98.5%
IL-17+ Cells 1.09%
IL-17+ Cells 0.277%
IL-17 PerCP Cy5.5
d
Cells
Doublet Exclusion
CD3/B220/Ter119 Dump Channel
Ly6G-Ly6C+ Monocytes
80.8
97.4
48.5
12.5
CD3 / B220 / Ter119 PE
Ly6C PerCP Cy5.5
Ly6G APC

Supplementary Fig. 3. Gating strategies (a) Splenocytes were gated (FSC-A vs. SSC-A) and then live cells determined by their uptake of the fixable live/dead cell stain before gating for singlets (FSC-H vs. FSC-A) prior to assessing expression of $CD19^{+}$ $IL\text{-}10^{+}$ B cells (Fig. 2f), with reference to relevant isotype controls. (**b**) Following exclusion of dead cells/cell debris and gating of lymphocytes (by forward scatter versus side scatter), cell doublets were excluded prior to subsequent gating of $CD3^{+}$ and $CD3^{+}IL\text{-}10^{+}$ populations relative to relevant FMO controls (Fig. 5g). (**c**) Following exclusion of MLN dead cells/cell debris and gating of lymphocytes (by forward scatter versus side scatter), cell doublets were excluded prior to subsequent gating of $IL\text{-}17^{+}$ populations relative to the relevant isotype control (Fig. 6c). (**d**) Gating strategy for identifying bone marrow (BM) $Ly6G^{-}Ly6C^{+}$ monocytes (Fig. 6e) following exclusion of dead cells/cell debris by forward scatter versus side scatter and exclusion of cells doublets. Labelling of BM cells with a cocktail of PE-labelled antibodies specific for CD3, B220 and Ter119 allowed exclusion of lymphocytes and erythroid cell populations using a dump channel, and monocytes were identified by labelling with antibodies against Ly6C (PerCP Cy5.5) and Ly6G (APC) as shown.

Supplementary Table 1. Bacterial abundance of the Colon samples (from Fig. 1b).

Bacteria	Naïve	PBS	ES-62
Firmicutes	63.367	28.901	50.344
Clostridia	57.453	25.434	44.993
Clostridiales	57.380	25.393	44.873
Bacteroidetes	34.556	64.198	48.062
Bacteroidia	34.207	64.081	47.770
Bacteroidaceae	24.700	60.203	38.278
Clostridiaceae	21.931	9.504	17.326
Clostridium	21.903	9.492	17.300
Lachnospiraceae	15.064	7.304	12.544
Ruminococcaceae	11.578	4.675	8.504
Alistipes putredinis	7.350	2.054	6.872
Rikenellaceae	7.350	2.054	6.872
Ruminococcus	6.670	2.374	4.265
Roseburia	5.970	3.266	6.149
Eubacteriaceae	5.483	2.475	4.078
Eubacterium	5.059	2.369	3.840
Clostridium methylpentosum	3.896	1.803	2.967
Marvinbryantia formatexigens	3.495	1.719	2.558
unclassified (derived from Lachnospiraceae)	3.495	1.719	2.558
Erysipelotrichi	3.196	1.500	2.175
unclassified (derived from Clostridiales)	3.120	1.346	2.280
Roseburia inulinivorans	3.103	1.459	3.105
Roseburia intestinalis	2.867	1.806	3.044
Clostridium nexile	2.773	1.054	2.175
Bacteroides fragilis	2.765	2.147	3.137
Clostridium scindens	2.650	1.215	2.386
Bacilli	2.538	1.900	3.058
Clostridium hathewayi	2.429	1.042	1.894
Eubacterium rectale	2.299	0.997	1.258
Lactobacillales	2.260	1.770	2.869
Blautia	2.048	0.864	1.420
Bacteroides uniformis	1.991	4.644	5.045
Butyrivibrio	1.937	0.805	1.255
Coprococcus	1.859	0.740	1.194
Bacteroides vulgatus	1.819	21.460	4.413
Clostridium bolteae	1.798	0.678	1.116
Faecalibacterium	1.761	0.946	1.998
Faecalibacterium prausnitzii	1.761	0.946	1.998

Holdemania	1.706	0.751	1.269
Holdemania filiformis	1.706	0.751	1.269
Bacteroides ovatus	1.684	1.279	2.243
Bacteroides sp. D20	1.610	3.341	3.557
Lactobacillaceae	1.570	1.213	1.796
Bacteroides capillosus	1.524	0.584	1.200
Clostridium sp. M62/1	1.472	0.536	1.225
unclassified (derived from Erysipelotrichaceae)	1.348	0.691	0.806
Proteobacteria	1.338	6.530	0.952
Clostridium difficile	1.308	0.556	0.929
Porphyromonadaceae	1.291	1.238	1.553
Butyrivibrio crossotus	1.280	0.404	0.737
Dorea	1.246	0.544	0.875
Ruminococcaceae bacterium D16	1.199	0.466	0.804
unclassified (derived from Ruminococcaceae)	1.199	0.466	0.804
Bacteroides sp. 1_1_6	1.193	1.194	1.493
Bacteroides sp. 2_2_4	1.182	1.377	1.404
Blautia hydrogenotrophica	1.174	0.506	0.633
Bacteroides xylanisolvens	1.123	0.891	1.523
Parabacteroides	1.116	1.035	1.198
Coprococcus comes	1.115	0.434	0.801
Clostridium saccharolyticum	1.089	0.489	0.856
unclassified (derived from Clostridiales)	1.066	0.479	0.851
Clostridiales bacterium 1_7_47FAA	1.058	0.471	0.836
Anaerotruncus	1.048	0.544	0.827
Anaerotruncus colihominis	1.048	0.544	0.827
Bacteroides sp. 4_3_47FAA	0.964	9.752	2.270
Eubacterium eligens	0.874	0.400	0.737
Blautia hansenii	0.874	0.358	0.787
Clostridium phytofermentans	0.862	0.436	0.799
Erysipelotrichaceae bacterium 5_2_54FAA	0.860	0.359	0.430
Prevotellaceae	0.853	0.584	1.064
Bacteroides sp. D2	0.807	0.650	1.030
Eubacterium ventriosum	0.777	0.451	0.809
Dorea formicigenerans	0.762	0.310	0.462
Coprococcus eutactus	0.744	0.306	0.392
Bacteroides caccae	0.723	0.632	1.059
Clostridium leptum	0.716	0.238	0.489
Bacteroides dorei	0.707	4.120	1.363
Bacteroides finegoldii	0.696	0.622	0.948
Clostridium proteoclasticum	0.657	0.400	0.518
Bacteroides intestinalis	0.647	0.799	0.759

Bacteroides sp. D22	0.604	0.474	0.799
Clostridium sp. HGF2	0.600	0.292	0.476
Bacteroides thetaiotaomicron	0.563	0.609	0.762
Deltaproteobacteria	0.518	0.214	0.412
Clostridium asparagiforme	0.510	0.380	0.634
Clostridium hylemonae	0.500	0.207	0.449
Dorea longicatena	0.483	0.234	0.414
Ethanoligenens	0.481	0.111	0.352
Ethanoligenens harbinense	0.481	0.111	0.352
Desulfovibrionales	0.469	0.190	0.372
Desulfovibrionaceae	0.462	0.185	0.369
Eubacterium hallii	0.449	0.212	0.325
Bacteroides sp. 2_1_33B	0.445	0.433	0.387
Gammaproteobacteria	0.443	0.898	0.251
Bacteroides pectinophilus	0.442	0.240	0.460
Clostridium sp. L2-50	0.432	0.124	0.225
Bacteroides sp. 20_3	0.417	0.411	0.380
Subdoligranulum	0.416	0.226	0.254
Subdoligranulum variabile	0.416	0.226	0.254
Anaerofustis	0.413	0.095	0.226
Anaerofustis stercorihominis	0.413	0.095	0.226
Desulfovibrio	0.389	0.161	0.316
Bacteroides coprocola	0.383	0.344	0.544
Parabacteroides distasonis	0.381	0.357	0.268
Eubacterium saburreum	0.367	0.154	0.409
Anaerostipes	0.363	0.116	0.265
Anaerostipes caccae	0.363	0.116	0.265
Actinobacteria	0.335	0.187	0.318
Parabacteroides sp. D13	0.328	0.270	0.315
Enterococcaceae	0.320	0.342	0.689
Clostridium sp. SS2/1	0.315	0.159	0.183
Bacteroides sp. 9_1_42FAA	0.308	1.163	0.503
Bacillales	0.278	0.129	0.188
Bacteroides sp. 3_1_23	0.245	0.154	0.229
Parabacteroides johnsonii	0.230	0.172	0.299
Bacteroides stercoris	0.216	0.438	0.351
Bacteroides cellulosilyticus	0.207	0.191	0.298
Eubacterium dolichum	0.205	0.133	0.108
Flavobacteria	0.204	0.064	0.159
Enterococcus faecalis	0.203	0.277	0.539
Bacteroides plebeius	0.199	0.201	0.260
Erysipelotrichaceae bacterium 3_1_53	0.197	0.159	0.203

Eubacterium cellulosolvens	0.196	0.060	0.155
Bacteroides sp. 3_1_33FAA	0.185	0.708	0.430
Bacteroides eggerthii	0.184	0.291	0.294
Negativicutes	0.181	0.067	0.118
Selenomonadales	0.181	0.067	0.118
Aerococcaceae	0.180	0.059	0.115
Parabacteroides merdae	0.176	0.235	0.316
Abiotrophia	0.175	0.056	0.094
Abiotrophia defectiva	0.175	0.056	0.094
Flavobacteriaceae	0.173	0.053	0.121
Betaproteobacteria	0.167	0.121	0.122
Bacteroides sp. 2_1_16	0.159	0.280	0.247
Streptococcaceae	0.154	0.134	0.220
Coriobacteriales	0.152	0.115	0.201
Enterobacteriales	0.151	0.826	0.130
Streptococcus	0.151	0.129	0.210
Burkholderiales	0.139	0.107	0.102
Alphaproteobacteria	0.138	0.038	0.096
Bacteroides sp. D1	0.136	0.138	0.253
Clostridium cellulovorans	0.134	0.053	0.054
Desulfovibrio sp. 3_1_syn3	0.128	0.051	0.095
Porphyromonas	0.124	0.103	0.205
Bacteroides sp. 2_1_7	0.123	0.121	0.084
Desulfovibrio desulfuricans	0.123	0.035	0.086
Bacteroides sp. 1_1_14	0.122	0.085	0.167
Bacillaceae	0.122	0.066	0.066
Bacteroides sp. 3_1_19	0.118	0.128	0.058
Veillonellaceae	0.114	0.039	0.076
Actinomycetales	0.099	0.031	0.052
Prevotella buccalis	0.089	0.086	0.164
Prevotella ruminicola	0.089	0.054	0.124
Bacillus	0.089	0.043	0.055
Enterococcus faecium	0.087	0.058	0.118
Porphyromonas gingivalis	0.087	0.053	0.077
Clostridium botulinum	0.087	0.046	0.064
Shuttleworthia	0.086	0.047	0.098
Shuttleworthia satelles	0.086	0.047	0.098
Prevotella oris	0.085	0.038	0.062
Staphylococcaceae	0.085	0.024	0.043
Staphylococcus	0.082	0.023	0.041
Bacteroides coprophilus	0.081	0.127	0.081
Bacteroides sp. 2_1_22	0.081	0.060	0.104

Oribacterium	0.081	0.058	0.073
Bifidobacteriales	0.079	0.038	0.063
Peptococcaceae	0.079	0.033	0.053
Bifidobacterium	0.078	0.029	0.035
Clostridium perfringens	0.078	0.023	0.041
Catenibacterium	0.075	0.033	0.057
Catenibacterium mitsuokai	0.075	0.033	0.057
Lawsonia	0.073	0.024	0.053
Lawsonia intracellularis	0.073	0.024	0.053
Epsilonproteobacteria	0.071	5.256	0.069
Prevotella tannerae	0.071	0.033	0.074
Prevotella buccae	0.070	0.042	0.065
Acidaminococcaceae	0.068	0.028	0.041
Campylobacterales	0.066	5.253	0.066
Desulfovibrio vulgaris	0.066	0.042	0.049
Rhizobiales	0.065	0.009	0.018
Thermoanaerobacterales	0.064	0.037	0.114
Prevotella sp. oral taxon 472	0.064	0.020	0.065
Desulfovibrio piger	0.062	0.027	0.067
Eubacterium biforme	0.062	0.016	0.036
Prevotella timonensis	0.058	0.030	0.068
Pasteurellales	0.057	0.015	0.036
Oribacterium sp. oral taxon 078	0.056	0.038	0.039
unclassified (derived from Bacteroidetes)	0.056	0.015	0.029
Eubacterium limosum	0.053	0.038	0.071
Bacteroides sp. 3_2_5	0.050	0.117	0.143
Paludibacter	0.050	0.101	0.150
Paludibacter propionicigenes	0.050	0.101	0.150
Eggerthella	0.048	0.048	0.084
Fusobacteria	0.048	0.027	0.045
Collinsella	0.048	0.020	0.043
Prevotella disiens	0.048	0.019	0.052
Photorhabdus	0.048	0.017	0.058
Photorhabdus luminescens	0.048	0.017	0.057
Campylobacteraceae	0.046	0.033	0.029
Sphingobacteria	0.045	0.029	0.066
Prevotella bryantii	0.045	0.020	0.041
Mitsuokella	0.045	0.009	0.013
Prevotella melaninogenica	0.044	0.037	0.080
Campylobacter	0.044	0.031	0.027
Prevotella sp. oral taxon 299	0.044	0.014	0.040
Clostridiales Family XI. Incertae Sedis	0.044	0.010	0.029

Cytophagia	0.044	0.009	0.037
Peptostreptococcaceae	0.042	0.010	0.049
Eubacterium siraeum	0.041	0.046	0.057
Spirochaetes	0.041	0.027	0.030
Sphingobacteriaceae	0.040	0.027	0.064
Campylobacter jejuni	0.038	0.021	0.018
Prevotella bivia	0.037	0.070	0.029
Streptococcus pneumoniae	0.037	0.024	0.028
Cyanobacteria	0.036	0.024	0.044
Paenibacillaceae	0.032	0.020	0.045
Streptococcus equinus	0.030	0.041	0.040
Paenibacillus	0.030	0.019	0.034
Escherichia	0.029	0.692	0.026
Prevotella copri	0.029	0.034	0.053
Clostridium hiranonis	0.029	0.021	0.052
Clostridium acetobutylicum	0.028	0.026	0.011
Alkaliphilus	0.028	0.013	0.026
Cellulosilyticum	0.028	0.009	0.076
Clostridium lentocellum	0.028	0.009	0.076
Escherichia coli	0.026	0.622	0.022
Synergistetes	0.026	0.010	0.018
Oribacterium sinus	0.025	0.020	0.034
Fusobacterium	0.025	0.015	0.033
Chlorobi	0.024	0.016	0.038
Clostridium spiroforme	0.023	0.021	0.022
Streptococcus suis	0.023	0.020	0.047
Carnobacteriaceae	0.023	0.016	0.037
Gramella	0.023	0.013	0.018
Alteromonadales	0.023	0.012	0.020
Clostridium bartlettii	0.023	0.010	0.024
Porphyromonas uenonis	0.021	0.016	0.037
Granulicatella	0.021	0.015	0.035
Helicobacteraceae	0.020	5.220	0.037
Helicobacter	0.020	5.216	0.033
Prevotella marshii	0.020	0.026	0.035
Desulfitobacterium	0.019	0.015	0.021
Clostridium carboxidivorans	0.019	0.013	0.011
unclassified (derived from Flavobacteriales)	0.019	0.009	0.025
Rhodobacterales	0.019	0.009	0.016
Coprobacillus	0.017	0.016	0.025
Coprobacillus sp. D7	0.017	0.016	0.025
Sphingobacterium	0.017	0.015	0.029

Pedobacter	0.017	0.011	0.029
Cryptobacterium	0.016	0.016	0.030
Prevotella oralis	0.016	0.015	0.015
Chloroflexi	0.016	0.013	0.017
Slackia	0.016	0.011	0.026
Prevotella sp. oral taxon 317	0.015	0.015	0.036
Selenomonas	0.015	0.015	0.020
Prevotella bergensis	0.015	0.009	0.036
Clostridium beijerinckii	0.015	0.008	0.035
Porphyromonas endodontalis	0.013	0.031	0.086
Helicobacter hepaticus	0.000	4.952	0.013
Deferribacteres	0.000	0.015	0.022
Clostridium kluyveri	0.000	0.013	0.019
